# Colobronchial fistula presenting with persistent pneumonia in a patient with Crohn's disease: a case report

**DOI:** 10.1186/1757-1626-2-9114

**Published:** 2009-11-30

**Authors:** Turki AlAmeel, D Alex MacLean, Ryan MacDougall

**Affiliations:** 1 Division of Gastroenterology, The University of Western Ontario, London, ON, Canada; 2 Department of Medicine, Dalhousie University, Halifax, Nova Scotia, Canada; 3 Department of Radiology, Dalhousie University, Halifax, Nova Scotia, Canada

## Abstract

Colobronchial fistula is an uncommon complication of Crohn's Disease. It is also rarely diagnosed on first presentation. We describe a case of colobronchial fistula and recurrent pneumonia in a patient with Crohn's Disease.

A 52-year-old gentleman with a history of Crohn's Disease presented with cavitating left lower lobe pneumonia that did not resolve despite a one month course of antibiotics. A computed tomography of the thorax confirmed the presence of a cavitating left lower lobe pneumonia. A subsequent abdominal computed tomography revealed a fistulous communication between the colon at the splenic flexure and the left bronchial space. The patient underwent surgery and a fibrous tract was visualized from the splenic flexure to the left lung. Medical treatment was continued with a six week course of antibiotics and the patient was doing well 12 weeks after surgery.

There have been few case reports of colobronchial fistula with a clinical picture similar to this case.

## Introduction

Crohn's disease is a chronic granulomatous disease of the gastrointestinal tract that is commonly complicated by fistulous communication between the inflamed bowel and adjacent organs or the skin.

Perianal fistulas are the most common [1]. Other frequent types are enteroenteric, enterovesical, and enterocutaneous fistulas.

There are few reports of patients with colobronchial fistula presenting with chronic pneumonia.

In this paper, we describe a case of colobronchial fistula secondary to Crohn's disease presenting with cavitating pneumonia unresponsive to antibiotics.

## Case presentation

A 52-year-old Canadian Caucasian man with history of Crohn's disease was refereed to our hospital for management of a persistent left lower lobe pneumonia. He had dyspnea and cough productive of fecal smelling sputum for one month. The symptoms were gradually worsening and he sustained a 13 kg weight loss over a period of one year. He had no diarrhea or anorexia. His clinical condition failed to improve despite oral antibiotic therapy with Co-trimoxazole, Ciprofloxacin and Fluconazole.

His relevant past medical history included a remote cecal-sigmoidal anastomosis without resection several years before this admission, and a motor vehicle accident that left him with mild cognitive dysfunction.

On admission, he had a temperature of 36.5°C, heart rate of 100 beats per minute, blood pressure of 110/70 mmHg, and an oxygen saturation of 94% on room air. Chest examination revealed tactile vocal fremitus and bronchial breath sounds over the left base. Laboratory data showed white blood cell count of 8.3*109/L, hemoglobin 96 g/l, MCV 74.7 fL, and platelets of 637*109/L. Chest X-ray showed a cavitating left lower lobe pneumonia. Sputum cultures prior to admission grew multiple organisms including *Escherichia coli*, *Candida *species, and *Stenotrophomonas Maltophilia*.

**Figure 1:**
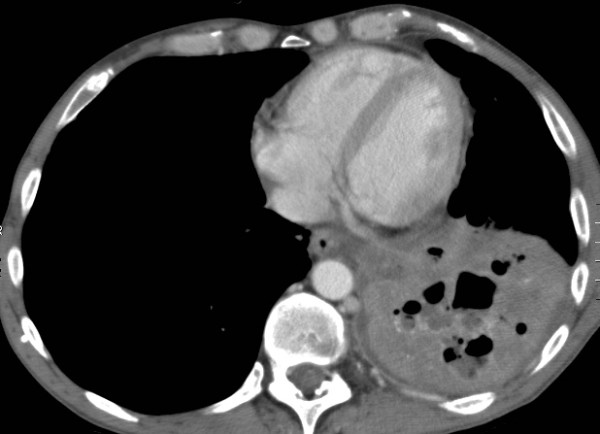
**Axial CT image shows a left lower lobe consolidation**. Irregular fluid- and gas-containing collections are seen in keeping with cavitation.

**Figure 2:**
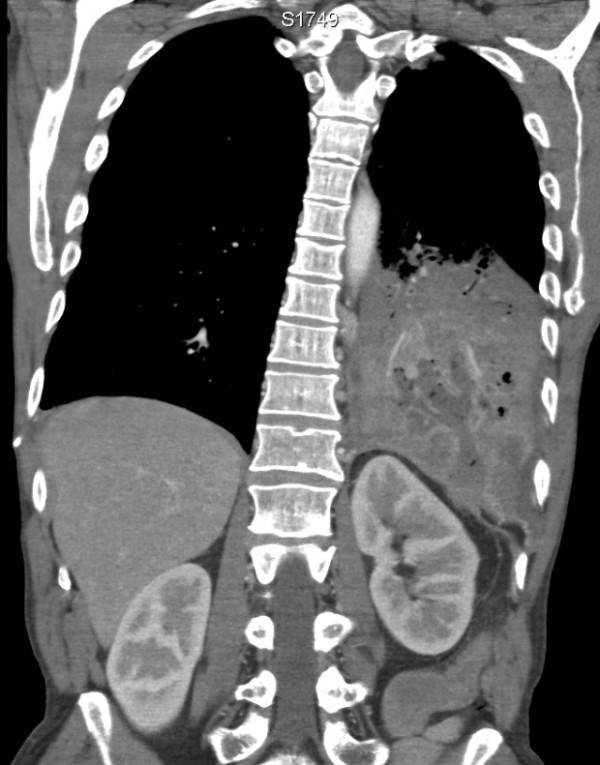
**Coronal CT image shows the cavitating left lower lobe pneumonia**. The fluid- and gas-containing collection transgresses the diaphragm and enters the retroperitoneal space. Although not shown here, this collection is contiguous with an abnormally thickened splenic flexure.

**Figure 3:**
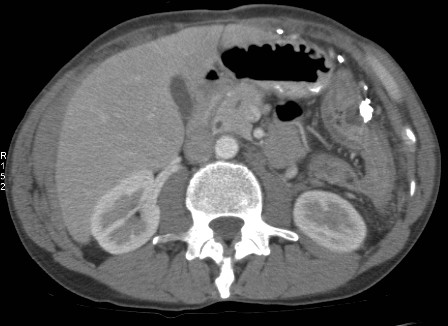
**Axial CT image shows the abnormally thickened splenic flexure with surrounding hazy stranding in keeping with inflammation**. The colobronchial fistula arises from this loop.

The patient was admitted to our hospital and started on Meropenem intravenously with no significant improvement. Bronchoscopy showed bronchial edema with no obstructing lesion. Contrast-enhanced CT thorax confirmed the presence of a cavitating left lower lobe pneumonia (Figure 1). There was also a suggestion that the pneumonia had transgressed the diaphragm and so an abdominal CT was arranged. The subsequent abdominal CT revealed a fistulous communication between the colon at the splenic flexure and the left bronchial space (Figure 2). The CT also showed evidence of segmental colitis involving the splenic flexure (Figure 3).

The patient underwent surgery with resection of the cecum and descending colon, preserving the previous cecal-sigmoid anastomosis. A fibrous tract was visualized extending from the splenic flexure, behind the spleen, and terminating in the left lung.

The patient was treated with a 6 week course of oral Amoxicillin/Clavulinic acid and was doing well when he was seen in clinic 12 weeks after surgery.

## Discussion

Internal fistulas are common in patients with Crohn's disease, occurring in 22% of patients [1]. However colobronchial fistula are rare with only few reported cases in the literature [2-7].

The case we describe had several features similar to the previously reported cases; a common presentation was left lower lobe pneumonia resistant to antibiotic treatment in a patient with either a history of Crohn's disease or chronic gastrointestinal symptoms [6]. An important clue to the diagnosis is the production of sputum with feculent odor. Another characteristic feature of the sputum is the growth of multiple organisms including gram negative bacteria, which is uncommon with community acquired pneumonia [8].

As in our case, patients usually present with respiratory symptoms with little or no signs of active colitis. This is a main reason for the delay in investigating the colon and thus making the diagnosis.

In all cases, colobronchial fistula extended from the splenic flexure in the colon to the lower lobe of the left lung. This is likely due to the anatomical proximity between the two structures.

This diagnosis is greatly facilitated by contrast-enhanced computed tomography. In previous reports barium enema was commonly used. In our case, the diagnosis was made with enhanced CT of the abdomen which revealed a clear communication between the colon and the left bronchial space. Most patients underwent surgical treatment, except in one case where the patient declined surgery [7].

In addition to colobronchial fistulas in patients with Crohn's disease, there have been reports of colobronchial fistulas due to colon cancer [9], tuberculosis [10] and radiation therapy [11].

## Conclusion

Our report prompts clinicians to consider the possibility of colobronchial fistula when they encounter a case of non-resolving left lower lobe pneumonia in a patient with underlying Crohn's disease.

## Competing interests

The authors declare that they have no competing interests.

## Authors' contributions

TA designed the study and drafted the manuscript.

AM wrote the clinical presentation and reviewed the literature.

RM choose the radiographic studies and commented on them.

All authors contributed to and approved the final manuscript.

## Consent

Written consent was obtained from the patient for publication of the case.
